# The cold-induced basic helix-loop-helix transcription factor gene *MdCIbHLH1 *encodes an ICE-like protein in apple

**DOI:** 10.1186/1471-2229-12-22

**Published:** 2012-02-15

**Authors:** Xiao-Ming Feng, Qiang Zhao, Ling-Ling Zhao, Yu Qiao, Xing-Bin Xie, Hui-Feng Li, Yu-Xin Yao, Chun-Xiang You, Yu-Jin Hao

**Affiliations:** 1 The State Key Laboratory of Crop Biology; National Research Center for Apple Engineering and Technology; College of Horticulture Science and Engineering, Shandong Agricultural University, Tai-An, Shandong 271018, China; 2 Yantai Academy of Agricultural Science, Yantai, Shandong 265500, China; 3 Shandong Institute of Pomology, Tai-An, Shandong 271000, China

## Abstract

**Background:**

Plant growth is greatly affected by low temperatures, and the expression of a number of genes is induced by cold stress. Although many genes in the cold signaling pathway have been identified in *Arabidopsis*, little is known about the transcription factors involved in the cold stress response in apple.

**Results:**

Here, we show that the apple bHLH (basic helix-loop-helix) gene *MdCIbHLH1 *(*Cold-Induced bHLH1*), which encodes an ICE-like protein, was noticeably induced in response to cold stress. The MdCIbHLH1 protein specifically bound to the MYC recognition sequences in the *AtCBF3 *promoter, and *MdCIbHLH1 *overexpression enhanced cold tolerance in transgenic *Arabidopsis*. In addition, the MdCIbHLH1 protein bound to the promoters of *MdCBF2 *and favorably contributed to cold tolerance in transgenic apple plants by upregulating the expression of *MdCBF2 *through the CBF (C-repeat-binding factor) pathway. Our findings indicate that MdCIbHLH1 functions in stress tolerance in different species. For example, ectopic *MdCIbHLH1 *expression conferred enhanced chilling tolerance in transgenic tobacco. Finally, we observed that cold induces the degradation of the MdCIbHLH1 protein in apple and that this degradation was potentially mediated by ubiquitination and sumoylation.

**Conclusions:**

Based on these findings, *MdCIbHLH1 *encodes a transcription factor that is important for the cold tolerance response in apple.

## Background

Apple (*Malus × domestica*) is one of the most important fruit crops worldwide, and low temperature is one of the major environmental factors affecting apple productivity in some commercial apple-growing regions. Apple trees require sufficient chilling during the winter period to resume normal growth and to develop their fruiting buds in the spring [1]. However, early spring chilling and late spring frosts that occur when growth has already resumed may damage apple trees [2,3]. Thus, improved tolerance to chilling may significantly increase apple production and economic benefit.

Plant responses to low temperatures are manifested at the physiological, molecular and biochemical levels [4]. At the molecular level, low temperatures induce a specific set of genes and proteins that mediate plant tolerance to cold stress. In response to cold temperatures, these genes and their products are involved in the protection of cellular machinery from stress-induced damage ([5,6]).

The ICE1-CBF-COR cold response pathway is one of the primary cold signaling modules that triggers the cold tolerance response in *Arabidopsis *([7-9]). *Arabidopsis COR *(cold-regulated) genes encode proteins that help protect against freezing stress [10]. The promoters of many of these *COR *genes contain copies of the C-repeat/dehydration-responsive element (CRT/DRE) ([11]). A family of AP2 domain-containing transcriptional activators, known as either CBF (CRT binding factor) [12,13] or DREB (DRE binding) proteins [14,15], bind to the CRT/DRE element and activate transcription. The genes encoding CBF transcription factors are also induced by cold, and the three *CBF *genes in *Arabidopsis *encoding DREB1B/CBF1, DREB1A/CBF3 and DREB1C/CBF2 have been found to participate in the cold-acclimation pathway [16-18]. Overexpression of the *DREB1A/CBF3 *and *DREB1B/CBF1 *genes in *Arabidopsis *increases the cold tolerance of transgenic plants [19]. Enhanced cold tolerance has also been observed in transgenic tobacco, tomato and rice expressing *CBF *genes [20-22]. In apple, overexpression of the *MbDREB1 *transcription factor gene increases plant tolerance to abiotic stress [23]. Additionally, heterologous overexpression of the *Prunus persica *CBF1 gene in apple enhanced freezing tolerance compared to the control M.26 plants [24].

The bHLH transcription factors are ubiquitously involved in the response of higher plants to various abiotic stresses, including cold (*AtICE1*, [9]), drought (*AtMYB2*, [25]) and high salinity (*AtNIG1*, [26]). ICE1 (Inducer of CBF3 Expression1) is a MYC-like basic helix-loop-helix transcription factor that activates *CBF3 *and *COR *genes in response to low temperatures in *Arabidopsis *[9]. ICE1 binds to the MYC recognition *cis*-elements (CANNTG) in the promoters of *CBF3 *gene, which triggers the expression of the CBF family members and subsequently enhances the expression of the CBF regulon [9,27]. A dominant mutation in *ICE1 *blocks the cold induction of CBF3 and reduces the expression of downstream genes [9]. Over-expression of *ICE1 *has been associated with enhanced chilling tolerance in *Arabidopsis*, cucumber and rice [28,29]. The degradation of the ICE1 protein strongly affects the expression of the genes encoding CBF3/DREB1A and CBF1/DREB1B along with the cold tolerance response [9]. In *Arabidopsis*, the degradation of ICE1 during acclimation to low temperatures is mediated by polyubiquitination at 26S proteasome pathway [30]. The ICE1 protein involved in cold acclimation is also modified by SUMO conjugation/deconjugation [31].

In this study, the bHLH gene *MdCIbHLH1 *(*COLD-INDUCED bHLH1*) was cloned based on its differential expression in response to cold stress. The specific binding of the protein encoded by *MdCIbHLH1 *to the promoters of *CBF *genes was verified with EMSA (Electrophoresis Mobility Shift Analysis) and ChIP-PCR (Chromatin immunoprecipitation). Subsequently, *MdCIbHLH1 *was transformed into *Arabidopsis*, apple and tobacco to characterize its functions in the cold response. The findings of this study indicate that the *MdCIbHLH1 *gene may be used to improve cold tolerance in apple and other crop species.

## Results

### Gene cloning, sequence analysis and MdCIbHLH1 subcellular localization

**Figure 1:**
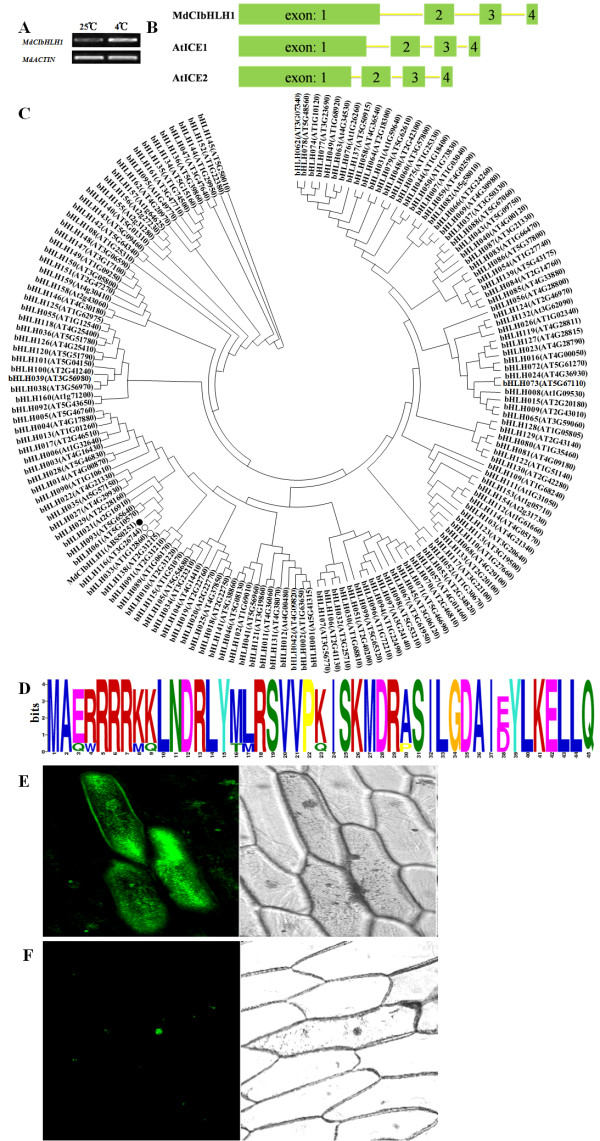
**Gene expression, sequence analysis and subcellular localization of MdCIbHLH1**.(A) Semi-quantitative RT-PCR analysis of *MdCIbHLH1 *at 25°C and 4°C. The apple tissue cultures were treated for 4 h. (B) Genomic structure of *MdCIbHLH1, AtICE1 *and *AtICE2*. (C) Phylogenetic tree containing MdCIbHLH1 and the bHLH proteins of *Arabidopsis*. The solid circle represents MdCIbHLH1, while empty cycles represent the *Arabidopsis *ICE1 and ICE2 bHLH transcription factors. (D) The bHLH domain is highly conserved in MdCIbHLH1 and the other bHLH TFs (AtICE1: AT3G26744; AtICE2: AT1G12860; CbICE1: AAS79350; GmICE1: ACJ39211; OsICE1: NP_001045272; PtICE1: ABF48720; PsICE2: ADK91821; GmICE4: ACJ39214; PsICE1: ABF48720; ThICE1: BAJ34291; GmICE2: ACJ39212; GmICE3: ACJ39213) proteins. The bit score indicates the information content for each position in the sequence. (E) Three onion epidermal cells producing GFP alone. (F) An onion epidermal cell producing the MdCIbHLH1-GFP fusion protein.

Within the publicly available apple GenBank (http://www.ncbi.nlm.nih.gov), there are a large number of ESTs exhibiting high similarity to *Arabidopsis *bHLH TF (transcription factors) from BLAST searches. Differential expression analysis was conducted with semi-quantitative RT-PCR to identify bHLH ESTs associated with cold induction in apple, and a differentially expressed EST was isolated using this methodology (Figure 1A). Subsequently, the full-length cDNA was cloned using the RACE technique following EST-based *in silico *cloning. Hereafter, the gene is referred to as *MdCIbHLH1 *(*Cold-Induced bHLH1*; GenBank accession number ABS50521).

Sequence analysis showed that three introns were present in the open reading frame (ORF) of *MdCIbHLH1 *along with four exons of the following lengths: 1113 bp, 230 bp, 169 bp and 84 bp (Figure 1B). The *MdCIbHLH1 *ORF was found to be 1596 bp in length and to encode a predicted protein containing 531 amino acid residues with a molecular weight of 57.4 kD.

The MEGA 4.0 program was used to construct phylogenetic trees for the bHLH proteins. For this analysis, all *Arabidopsis *bHLH proteins and MdCIbHLH1 were included and aligned. The MdCIbHLH1 protein clustered within the same clade as AtbHLH116 (AtICE1) and AtbHLH033 (AtICE2) (Figure 1C). In addition, genomic comparisons revealed that the *MdCIbHLH1, AtICE1 *and *AtICE2 *genes all contained four exons, indicating a similar genomic composition (Figure 1B).

The predicted MdCIbHLH1 protein was found to contain a conserved bHLH domain and a nuclear localization signal http://www.bioinfo.tsinghua.edu.cn/SubLoc/. The bHLH domain of MdCIbHLH1 exhibited high similarity to the bHLH domains of ICE proteins in other plant species (Figure 1D).

The presumed nuclear localization signal in MdCIbHLH1 was sufficient to direct a GFP fusion protein to the nucleus. The subcellular localization of MdCIbHLH1 was investigated by introducing a translational fusion between *MdCIbHLH1 *and *GFP *into onion epidermal cells using *Agrobacterium*-mediated transformation. Cells expressing the *MdCIbHLH1:GFP *fusion gene showed GFP fluorescence only in the nucleus (Figure 1F), while cells expressing *GFP *alone showed GFP fluorescence throughout the entire cell (Figure 1E). This result suggests that the MdCIbHLH1 protein subcellularly localizes to the nucleus.

### Expression of *MdCIbHLH1 *in different tissues and in response to cold stress

**Figure 2:**
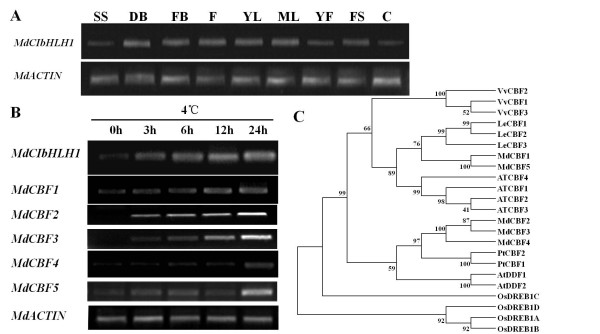
**Expression profiles of the *MdCIbHLH1 *gene in apple**. (A) Expression analysis of *MdCIbHLH1 *in SS (spring shoot), DB (dormant bud), FB (floral buds), F (flower), YL (young leaf), ML (mature leaf), YF (young fruit), FS (fruit skin) and C (callus). (B) Expression profiling of *MdCIbHLH1, MdCBF1, MdCBF2, MdCBF3, MdCBF4 *and *MdCBF5 *in 'Gala' apple tissue cultures in response to chilling treatment. Transcript levels were normalized to the expression of the apple *MdACTIN *gene. (C) Phylogenetic tree of CBF proteins in apple and other plant species.

*MdCIbHLH1 *transcript levels were analyzed in different tissues by semi-quantitative RT-PCR. The results showed that *MdCIbHLH1 *transcripts were constitutively present at different levels in the various tissues tested, including the spring shoot, dormant bud, floral bud, flower, young leaf, mature leaf, young fruit, fruit skin and callus (Figure 2A). To determine whether *MdCIbHLH1 *is induced by cold stress, its expression was analyzed by semi-quantitative RT-PCR in apple tissue cultures exposed to 4°C for different amounts of time. The results showed that *MdCIbHLH1 *expression was positively induced by the chilling treatment at 4°C (Figure 2B).

In *Arabidopsis*, AtCBFs are crucial components acting downstream of AtICE1 in the cold signaling pathway ([9]). In the apple genome, there are five CBF genes: *MdCBF1 *(U77378), *MdCBF2 *(AF074601), *MdCBF3 *(AF074602), *MdCBF4 *(NM_124578) and *MdCBF5 *(NM_101131). Phylogenetic analysis indicated that the five MdCBFs are highly similar to CBFs in other plant species, including grapevine, poplar, tomato, *Arabidopsis *and rice (Figure 2C). Interestingly, the expression of all five *MdCBFs *was positively induced by chilling treatment at 4°C following the increased expression of *MdCIbHLH1 *(Figure 2B), suggesting that *MdCIbHLH1 *may act upstream of the *MdCBFs*.

### MdCIbHLH1 regulates *AtCBFs *by binding to their promoters in *Arabidopsis*

**Figure 3:**
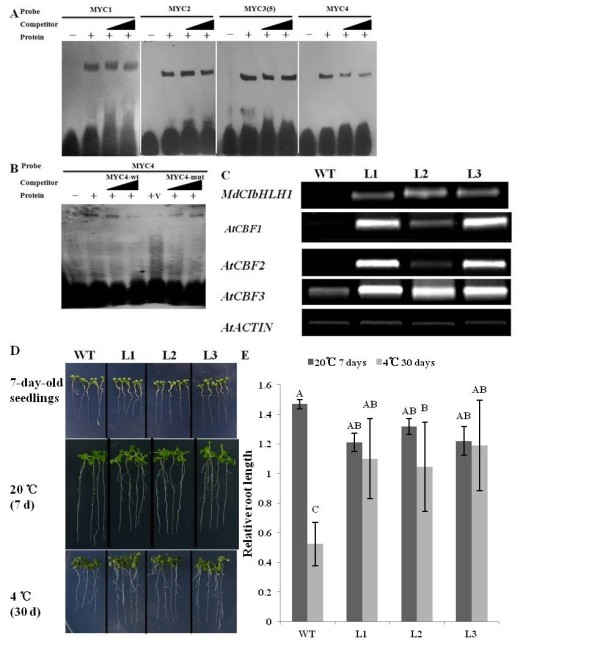
**MdCIbHLH1 binds to the *AtCBF3 *promoter and increased chilling tolerance in *Arabidopsis***. (A) Interaction between the MdCIbHLH1 protein and labeled MYC1, MYC2, MYC3(5) and MYC4 DNA fragments. (B) MdCIbHLH1 binding to the MYC4 DNA fragment. The labeled probes used in each experiment are indicated at the top of each panel. Triangles indicate increasing amounts of unlabeled oligonucleotides used for the competition analysis in (A) and (B), corresponding to a 100-fold and 400-fold excess of each probe. wt, probe with an intact element; mut, probe with a mutated element; v, protein product of the empty vector expressed in GS115. (C) The transcript levels of *MdCIbHLH1, AtCBF1, AtCBF2 *and *AtCBF3 *in transgenic *Arabidopsis *under control conditions. RNA was isolated from wild-type and transgenic seedlings. The *AtACTIN *gene was used as the loading control. WT, wild-type; L1, L2 and L3, transgenic *Arabidopsis *lines. (D) Chilling tolerance of WT, L1, L2 and L3 seedlings. (E) Relative root length of WT, L1, L2 and L3 seedlings exposed to chilling. Values indicated by the capital letters are not significant at p < 0.01, respectively, according to the Duncan's Multiple Range Test.

The similarity of MdCIbHLH1 to AtICE1 and AtICE2 indicates that it may function in a similar way to the AtICE proteins. To examine if the MdCIbHLH1 protein binds to MYC recognition sites associated with cold signaling, as AtICE1 does, EMSA was conducted to assess the DNA-binding capacity of MdCIbHLH1 to four CANNTG motifs, MYC1, MYC2, MYC3(5) and MYC4, in the *AtCBF3 *promoter [9]. Shifted bands were observed for MYC4 (Figure 3A). When 100-fold and 400-fold excess of non-labeled competitor DNA probe for MYC4 was added to the reaction, the presence of shifted bands was reduced (Figure 3A). In contrast, a mutated competitor had less of an effect on the binding between MdCIbHLH1 and the MYC4 fragment, compared to the wild-type competitor (Figure 3B). Therefore, the excess quantity of the non-labeled probe interfered with the specific binding of the labeled MYC4 promoter fragment. The non-specific competitor did not have this effect, indicating that the MdCIbHLH1 protein specifically binds to the MYC4 recognition site in the *AtCBF3 *promoter.

Band shifts were also observed for the reactions between MdCIbHLH1 and the MYC1, MYC2 and MYC3(5) probes (Figure 3A). However, the binding was not abolished by the addition of unlabeled wild-type MYC1, MYC2 and MYC3(5) competitor, respectively (Figure 3A), indicating that the binding of MdCIbHLH1 to MYC1, MYC2 and MYC3(5) was not as specific as the binding to MYC4. This is not the case for AtICE1, which specifically binds to all four recognition sites [9].

To further characterize the functions of the *MdCIbHLH1 *gene, a construct containing *MdCIbHLH1 *driven by the *CaMV 35S *promoter was genetically transformed into *Arabidopsis*. Three homozygous transgenic lines, L1, L2 and L3, were used for further investigation. Semi-quantitative RT-PCR showed higher levels of *MdCIbHLH1 *transcripts in the three lines than in the WT (wild type) control. In addition, the expression of *AtCBFs *increased remarkably in the 3 transgenic lines compared to the control (Figure 3C). Transgenic seedlings were used to examine chilling tolerance. Relative root lengths were measured after the chilling treatment. The results showed that at 20°C the ectopic expression of MdCIbHLH1 inhibited the root growth of the transgenic *Arabidiopsis *seedlings (Figure 3D-E). In contrast, at 4°C the relative root lengths of L1, L2 and L3 transgenic seedlings were 1.1, 1.04 and 1.19, respectively, while that of WT control was 0.52 (Figure 3D-E). Therefore, heterologous expression of *MdCIbHLH1 *conferred enhanced chilling tolerance in transgenic *Arabidopsis *seedlings.

### MdCIbHLH1 regulates cold tolerance via the CBF pathway in apple

**Figure 4:**
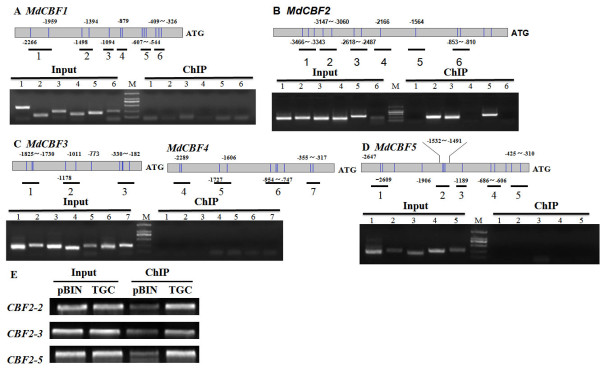
**ChIP analysis of *MdCIbHLH1 *in transgenic apple callus containing either the empty vector (pBIN) or the *MdCIbHLH1* gene (TGC) . ** (A-E) PCR products amplified from the promoters of *MdCBF1 *(A), *MdCBF2 *(B), *MdCBF3 *(C), *MdCBF4 *(C) and *MdCBF5 *(D) were size-fractionated by gel electrophoresis; representative gels are shown. Black lines represent the PCR products. Dilutions of input DNA were used as PCR templates to show linear amplification. (E) The ChIP product of transgenic apple callus. Input DNA was used as the control.

EMSA was used to verify the specific binding of MdCIbHLH1 to the MYC sequence in the *AtCBF3 *promoter. The promoters of the five *MdCBFs *also contain MYC recognition sites, and ChIP-PCR was conducted to determine whether MdCIbHLH1 protein binds to the promoters of the five *CBF *genes in apple. An *MdCIbHLH1-GFP *fusion construct driven by the *CaMV 35S *promoter was introduced into apple tissue cultures and calluses using *Agrobacterium*-mediated transformation. ChIP assays were performed using GFP antibodies. The ChIP product of transgenic callus transformed with an empty vector was used as a nonspecific control. After isolation of cross-linked chromatin, immunoprecipitated DNA was analyzed by PCR (Figure 4A-D). The results showed that MdCIbHLH1 specifically bound to the *MdCBF2 *promoter at three regions (Figure 4B). However, it failed to bind to the regions tested in the *MdCBF1, MdCBF3, MdCBF4 *and *MdCBF5 *promoters. The specific binding of MdCIbHLH1 to the *MdCBF2 *promoter was also observed in transgenic apple calluses (Figure 4E).

**Figure 5:**
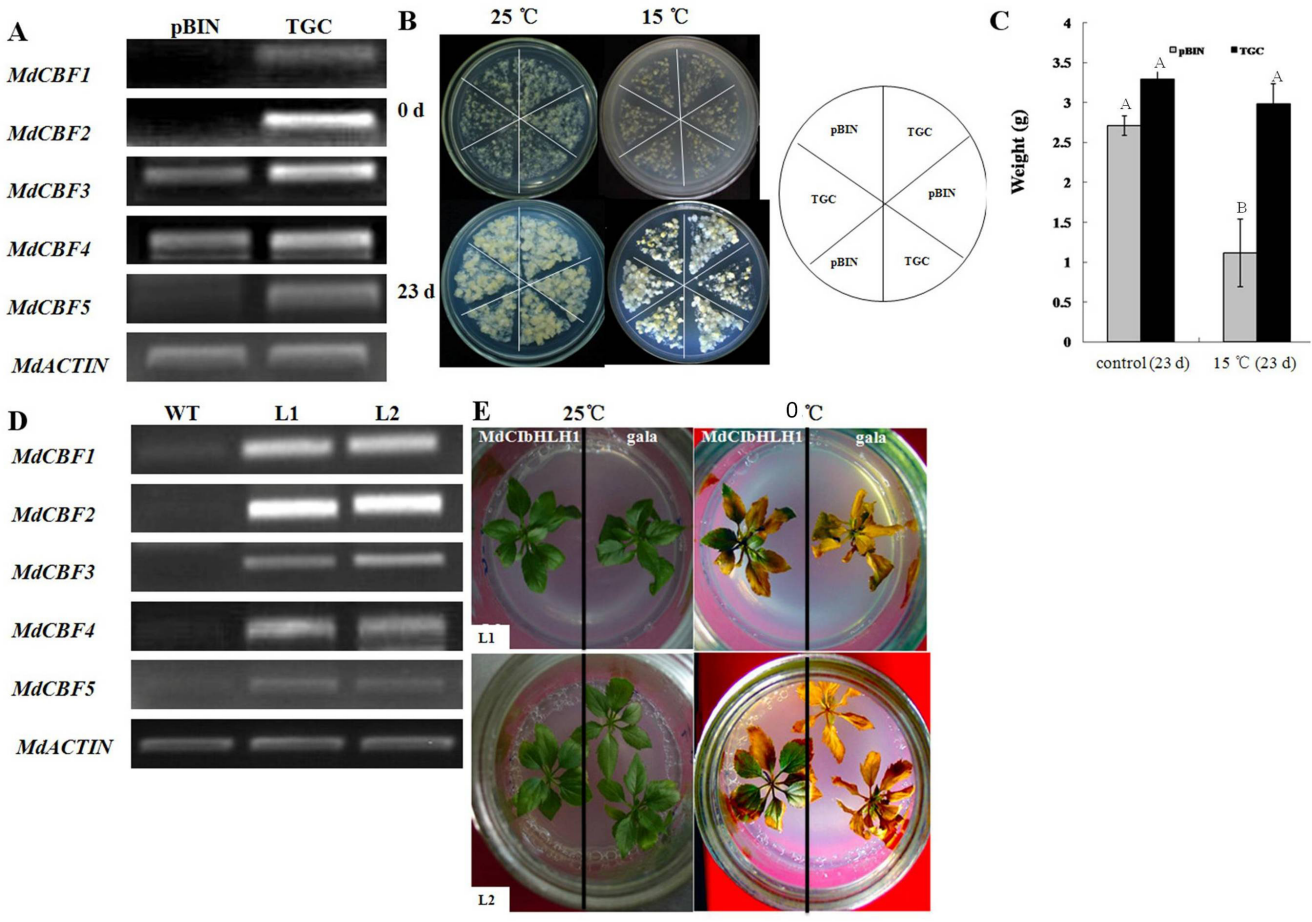
**Stress tolerance assay of *MdCIbHLH1 *in transgenic apple**. (A) Semi-quantitative RT-PCR analysis of *MdCIbHLH1, MdCBF1, MdCBF2, MdCBF3, MdCBF4 *and *MdCBF5 *in transgenic apple calluses containing either an empty vector (pBIN) or the *MdCIbHLH1 *gene (TGC). Differences in the starting quantity of template were normalized using *MdACTIN *expression. (B) Photographs of transgenic calluses under stress conditions. The upper panels represent calluses prior to stress analysis. The lower panels show calluses grown at 15°C for an additional 23 days. The cycle on the right is a sketch of callus distribution in a plate. (C) The weight increment of calluses that underwent stress treatment, shown in (B). Error bars indicate the standard errors from three independent experiments. (D) Semi-quantitative RT-PCR analysis of *MdCIbHLH1, MdCIbHLH1, MdCBF1, MdCBF2, MdCBF3, MdCBF4 *and *MdCBF5 *in transgenic and non-transgenic apple tissue culture seedlings. Differences in the starting quantity of template were corrected by normalizing to *MdACTIN *expression. (E) Plants of WT, L1 and L2 apple tissue culture seedlings were subjected to 0°C for 7 days and then grown for an additional 7 days under normal growth conditions. WT, wild type; L1 and L2, transgenic apple lines. Values indicated by the capital letters are not significant at p < 0.01, respectively, according to the Duncan's Multiple Range Test.

For the functional characterization of *MdCIbHLH1 *in apple callus, the transgenic callus line expressing the *MdCIbHLH1 *gene was referred to as the TGC (transgenic callus) line, and the transgenic callus line containing the empty vector *pBIN *was referred to as the pBIN control. Semi-quantitative RT-PCR showed higher transcript levels of *MdCIbHLH1 *in the TGC line than in the pBIN control, suggesting that the *MdCIbHLH1 *gene was overexpressed in the TGC line. In addition, the expression of *MdCBF1, MdCBF2, MdCBF3, MdCBF4 *and *MdCBF5 *was upregulated in the TGC line compared to the pBIN control (Figure 5A), further suggesting that *MdCIbHLH1 *functions upstream of the *MdCBFs *and upregulates their expression in part by directly binding to the *MdCBF2 *promoter. These results indicate that *MdCIbHLH1 *overexpression conferred enhanced chilling tolerance in the TGC line (Figure 5B-C).

The function of MdCIbHLH1 was also characterized in transgenic apple tissue cultures. Semi-quantitative RT-PCR showed high transcript levels of *MdCIbHLH1 *in 2 transgenic lines, L1 and L2, indicating that the *MdCIbHLH1 *gene was overexpressed in these transgenic lines. In addition, *MdCIbHLH1 *was found to upregulate the expression of *MdCBF1, MdCBF2, MdCBF3, MdCBF4 *and *MdCBF5 *(Figure 5D), further confirming that *MdCIbHLH1 *works upstream of the *MdCBFs*. WT and *MdCIbHLH1 *transgenic lines grown on MS media were exposed to 0°C temperature for 7 days. Following a 7-day recovery, the WT control was nearly dead, while the 2 transgenic lines were still alive (Figure 5E). *MdCIbHLH1 *overexpression therefore improved the tolerance to cold stress in transgenic apple lines.

### Overexpression of *MdCIbHLH1 *in tobacco

**Figure 6:**
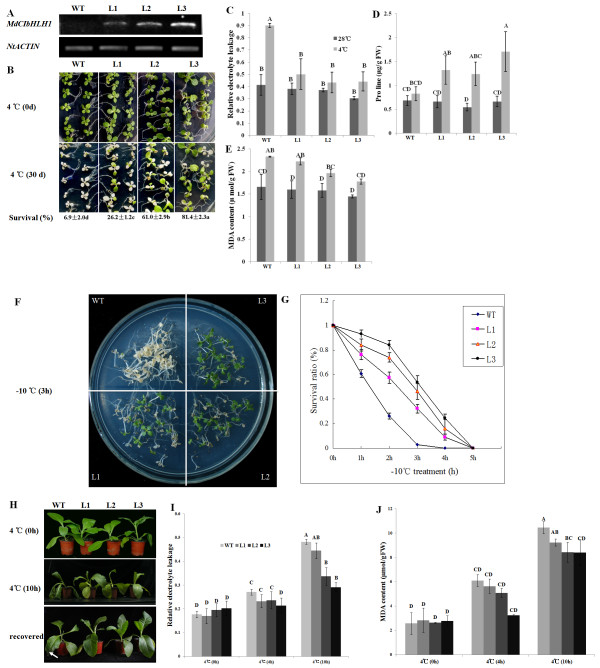
**Accumulation of *MdCIbHLH1 *and the effects of *MdCIbHLH1 *on chilling tolerance in tobacco**. (A) The transcript levels of *MdCIbHLH1 *in transgenic tobacco. The *NtACTIN *gene was used as a loading control. WT, wild-type; L1, L2 and L3, transgenic tobacco lines. (B) Chilling tolerance of WT, L1, L2 and L3 seedlings. (C-E) Relative electrolyte leakage (C), proline (D) and MDA contents (E) of WT, L1, L2 and L3 seedlings exposed to chilling. (F) Freezing tolerance of WT, L1, L2 and L3 seedlings. (G) The survival ratios of WT, L1, L2 and L3 seedlings exposed to freezing treatment. (H) Chilling tolerance of WT, L1, L2 and L3 adult tobacco plants. The arrow indicates the necrotic leaf margin. (I-J) Relative electrolyte leakage (I) and MDA content (J) in tobacco plants that underwent chilling treatment. Values indicated by the capital letters are not significant at *p *< 0.01, according to the Duncan's Multiple Range Test.

To examine the function of *MdCIbHLH1 *in another crop, transgenic tobacco plants heterologously expressing the *MdCIbHLH1 *gene were obtained. Semi-quantitative RT-PCR showed that the *MdCIbHLH1 *gene was heterologously expressed in three transgenic tobacco lines, L1, L2 and L3, at different levels (Figure 6A). Fifteen-day-old seedlings were used to examine tolerance to chilling stress. After maintaining the seedlings at 4°C for 30 days, their survival rate was determined. The results showed that the survival ratios of L1, L2 and L3 seedlings were 26.2 ± 1.2%, 61 ± 2.9% and 81.4 ± 2.3%, respectively, while that of the WT control was 6.9 ± 2% (Figure 6B). In addition, the three transgenic lines produced much more protectant proline but exhibited reduced injury, as indicated by the reduced MDA content and electrolyte leakage under chilling conditions compared to WT (Figure 6C-E). Heterologous *MdCIbHLH1 *expression therefore conferred enhanced chilling tolerance to transgenic tobacco plants. Fifteen-day-old seedlings were also used to assess freezing tolerance. The results showed that ectopic expression of *MdCIbHLH1 *noticeably enhanced the freezing tolerance of L1, L2 and L3 seedlings (Figure 6F-G). The survival ratios of the three transgenic lines were positively correlated with the expression levels of *MdCIbHLH1*, indicating that their enhanced tolerance was derived from the ectopic expression of *MdCIbHLH1*.

Adult plants were also used to assess chilling stress tolerance. The results showed that L1, L2 and L3 plants exhibited enhanced tolerance to chilling compared to WT plants (Figure 6H). After 4°C treatment for 10 h, WT plants started to wilt, while transgenic plants were near normal in appearance. Following a 2-h recovery under normal conditions, WT plants showed necrosis at the leaf margin, while the three transgenic lines completely recovered (Figure 6H). In addition, transgenic plants produced much less MDA than WT plants and therefore had less injury in their membrane systems, as indicated by the reduced electrolyte leakage relative to the WT control under chilling stress (Figure 6I-J).

Taken together, these data indicate that *MdCIbHLH1 *is involved in chilling tolerance in various plant species.

### MdCIbHLH1 protein is modified by ubiquitination and sumoylation

**Figure 7:**
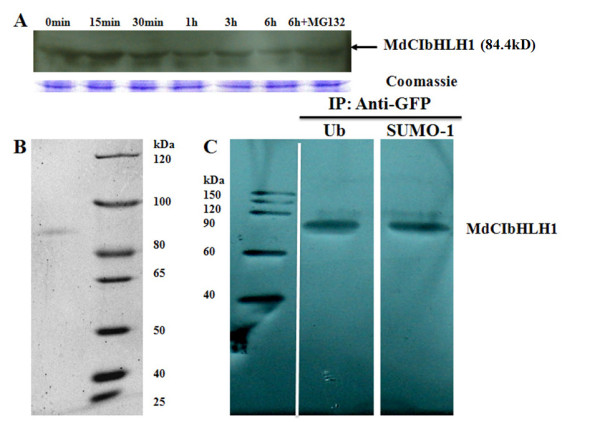
**Cold-induced degradation and post-translational modification of MdCIbHLH1**. (A) Detection of MdCIbHLH1-GFP protein by Western blot analysis. TGC calluses were maintained at 15°C for the cold treatment. MdCIbHLH1-GFP protein levels were analyzed using anti-GFP. Nonspecific Coomassie blue staining of a duplicate gel shows that similar amounts of proteins were loaded. (B) Detection of immunoprecipitated MdCIbHLH1-GFP protein by SDS/PAGE. (C) In vivo detection of ubiquitinated and sumoylated MdCIbHLH1-GFP in TGC calluses.

To determine the stability of the MdCIbHLH1-GFP fusion protein under cold stress, immunoblots were conducted using transgenic apple calluses. The results showed that MdCIbHLH1 protein gradually degraded to very low levels upon exposure to cold stress for 6 h. However, MG132, a 26S proteasome-specific protease inhibitor, remarkably suppressed the cold-induced protein degradation (Figure 7A), suggesting that the ubiquitination-associated proteasome is involved in this process. Therefore, in addition to cold-induced transcription, cold temperatures may also affect MdCIbHLH1 activity by modulating its protein abundance at a posttranslational level.

It has been reported that AtICE1 protein degrades at low temperatures through a ubiquitin-proteasome pathway [30]. In addition, sumoylation may function as a ubiquitin antagonist [31]. To determine if MdCIbHLH1 protein is modified by ubiquitination or sumoylation, MdCIbHLH1-GFP protein was immunoprecipitated using anti-GFP antibody in the TGC line (Figure 7B). The precipitated proteins were detected with anti-ubiquitin and anti-SUMO antibodies to determine the occurrence of ubiquitination and sumoylation modifications, respectively. Positive signals were detected for both antibodies (Figure 7C), suggesting that ubiquitination and sumoylation of the MdCIbHLH1 protein occur in vivo in TGC calluses.

## Discussion

In this study, the *MdCIbHLH1 *gene was isolated from apple, based on its differential expression in response to cold treatment. It encodes a bHLH TF. The bHLH TFs represent a family of proteins that contain a conserved bHLH domain, a motif involved in DNA binding [32]. The bHLH proteins regulate downstream genes through sequence-specific interactions at the promoter regions of these genes [32]. MdCIbHLH1 was found to have a highly similar gene structure and amino acid sequence to *Arabidopsis *ICE1 and ICE2 [9,33], suggesting that it is a putative apple ortholog of the ICE proteins. This is consistent with the fact that *MdCIbHLH1 *expression was induced by cold stress, as observed for ICE1 and ICE2 in *Arabidopsis *[9,33]. However, *MdCIbHLH1 *was found to have a longer sequence than *AtICE1 *and *AtICE2*, corresponding to the greater genome size of apple compared to *Arabidopsis*.

In *Arabidopsis*, ICE1 acts directly upstream of the CBFs by binding to the MYC recognition sites present in the promoters of the *CBF *genes, and this binding subsequently triggers the expression of the *CBFs/DREBs *regulon [9,27]. In *Arabidopsis*, AtICE1 and AtICE2 may directly regulate AtCBF3 and AtCBF1, respectively [9,33]. In this study, MdCIbHLH1 protein was found to bind to the MYC recognition site of the *AtCBF3 *and *MdCBF2 *promoters using EMSA and ChIP-PCR, further confirming that it functions similarly to ICE proteins. However, MdCIbHLH1 specifically bound to only one of the four putative MYC recognition sites in the promoter of *AtCBF3*, while AtICE1 binds to all 4 MYC sites. In apple, however, MdCIbHLH1 only bound to the *MdCBF2 *promoter. In accordance with the specific binding, the greatest increase in expression was observed for *MdCBF2*, but the expression of the other 4 *MdCBF *genes was also enhanced. This may be explained by either an incomplete conservation between MdCIbHLH1 and the AtICEs or the existence of other recognition sites in the promoters of the *MdCBF *genes. Additionally, MdCBF2 may regulate the expression of other *MdCBF *genes, as is the case for the AtCBFs in *Arabidopsis *[17].

The CBF cold responsive pathway is an important low-temperature gene network that contributes to cold tolerance ([7-9]). *DREB1B/CBF1, DREB1A/CBF3 *and *DREB1C/CBF2 *are induced by cold in *Arabidopsis*. Overexpression of the *DREB1A/CBF3 *and *DREB1B/CBF1 *genes increases cold tolerance in transgenic *Arabidopsis*, rapeseed, tobacco, tomato and rice [20-22]. In addition, the genes encoding CBF/DREB transcriptional activators are also associated with increased cold tolerance in apple [23,24].

A dominant mutation in *ICE1 *blocks the cold induction of the *CBF3 *regulon and impairs freezing and chilling tolerance [9]. In contrast, *ICE1 *overexpression increases cold tolerance relative to wild-type plants not only in *Arabidopsis *but also in other plants such as cucumber and rice [28,29]. In *Arabidopsis*, a recently identified positive regulator, ICE2, which is a bHLH transcription factor, participates in the response to freezing through the cold acclimation-dependent pathway [33]. In this study, *MdCIbHLH1 *overexpression resulted in elevated expression of the *CBF *genes and enhanced tolerance to cold stress. These data suggest that *MdCIbHLH1 *acts as a signal transduction component in the CBF pathway and is associated with cold tolerance in apple, as is the case for the *ICE *genes in *Arabidopsis *[9].

In *Arabidopsis*, cold induces AtICE1 proteolysis through a proteasome pathway [30]. It has been shown that the ubiquitination of AtICE1 is mediated by HOS1 both in vitro and in vivo [30]. In addition, AtSIZ1-mediated SUMO conjugation/deconjugation of AtICE1 is a critical process required for low temperature tolerance [31]. Unlike AtICE1, AtICE2 is not involved in ubiquitin-dependent proteolysis [33]. The results of the present study showed that the MdCIbHLH1 protein was degraded under cold conditions and that MdCIbHLH1 was modified through the ubiquitination and sumoylation pathways. However, the MdCIbHLH1 protein did not interact with AtHOS1 or AtSIZ1 (data not shown). Our results suggest that the degradation of MdCIbHLH1 protein depends on the presence of other ubiquitin and SUMO E3 ligases in apple.

The CBF pathway is also operational in trees [34]. It is well established that early spring chilling and late spring frosts that occur when growth has already resumed may cause damage to apple crops by direct chilling injury or by indirectly influencing pollination and fruit set efficiency [2,3]. The expression of *MdCIbHLH1 *was positively induced by cold treatment, suggesting that the *MdCIbHLH1 *gene may be involved in the protection of apple tissues from cold injury.

## Conclusions

In this study, we isolated an apple bHLH (basic helix-loop-helix) gene *MdCIbHLH1 *(*Cold-Induced bHLH1*). Its expression was noticeably induced in response to cold stress. It encodes an ICE-like protein. The degradation of the MdCIbHLH1 protein was induced by cold treatment and was potentially mediated by ubiquitination and sumoylation in apple. Furthermore, EMSA and ChIP-PCR verified that the MdCIbHLH1 protein specifically bound to the MYC recognition sequences in the promoters of *AtCBF3 *and *MdCBF2 *genes. As a result, the ectopic expression of *MdCIbHLH1 *enhanced cold tolerance in transgenic *Arabidopsis*, tobacco and apple seedlings or plants by upregulating the expression of *CBFs *genes through the CBF (C-repeat-binding factor) pathway. Therefore, MdCIbHLH1 functions in cold tolerance in a CBF-dependent way in different plant species. Reflecting its role in cold tolerance, MdCIbHLH1 may be an appropriate gene for overcoming cold stress and improving apple crop productivity under cold temperature conditions.

## Methods

### Plant materials

The in vitro tissue cultures of apple (*Malus domestic *cv. 'Royal Gala') were grown at 25 ± 1°C under a 16 h photoperiod for gene cloning and genetic transformation. 'Orin' apple calluses were cultured in the dark at 25 ± 1°C and used for genetic transformation. *Arabidopsis thaliana *(ecotype 'Columbia 0') was grown at 20 ± 1°C under a 16 h photoperiod and used for genetic transformation. Tobacco (*Nicotiana tobaccum *L. 'NC89') was grown at 25 ± 1°C under a 12 h photoperiod and used for genetic transformation. In vitro tissue cultures of 'Royal Gala' were used for expression analysis after cold treatment (4 ± 1°C).

### Semi-quantitative RT-PCR assays for gene expression

Total RNAs were extracted from apple and *Arabidopsis *samples using the RNAplant plus Reagent (Tiangen, China) and TRIzol Reagent (Invitrogen, USA), respectively, according to the manufacturers' instructions. First-strand cDNA was synthesized using the PrimeScript 1st Strand cDNA Synthesis Kit transcriptase (TaKaRa, Japan) according to the manufacturer's instructions.

The PCR reaction mixture contained 200 ng cDNA, 1 μl of each primer (10 mM), 2.5 μl 10× Taq DNA polymerase buffer containing Mg^2+ ^(20 mM), 2 μl deoxyribonucleotide (dNTP) (2.5 mM) and 0.25 μl 5 U/μl transTaq DNA polymerase (Trans, China) in a 25 μl reaction volume. For the semi-quantitative RT-PCR reactions, the numbers of reaction cycle were 25-30. *MdACTIN *was used to normalize cDNA loading. The following primers were used for the PCR reaction: MdCIbHLH1S: 5'-ATGGACGACAGGGAGGAC-3', MdCIbHLHlA: 5'-GGAGGAGGAAGAGTCCAC-3'; MdCBF1S: 5'-CGGTGTTTCGGGGTGTAAG-3'; MdCBF1A: 5'-AAGTGCCCAGCCAAATCC-3'; MdCBF2S: 5'-ATCCGACGGCCGAGATGGCA-3'; MdCBF2A: 5'-CCAAACTCCGCTGGCCGGAA-3'; MdCBF3S: 5'-AAGCGGCGATGATGAGAA-3'; MdCBF3A: 5'-CGTCATCGTTGCTTTCCAT-3'; MdCBF4S: 5'-GTCATTTTGGCATCCAGCAC-3'; MdCBF4A: 5'-TTGTTCCTCCTCACTCCTC-3'; MdCBF5S: 5'-GGAAGCAGCAGAACAGTTT-3'; MdCBF5A: 5'-CACGACATCATCGGCATTG-3'; *MdACTIN *was amplified using previously reported primers [35].

### Subcellular localization of MdCIbHLH1

The full-length coding region of *MdCIbHLH1 *was fused to the N-terminus of the *GFP *gene under the control of the *CaMV 35S *promoter. Transient expression of the *35S::MdCIbHLH1:GFP *fusion construct and the *GFP *control vector was introduced into onion epidermal cells using the *Agrobacterium*-mediated genetic transformation method. Transformed cells were cultured on MS media for 16-24 h in the dark and observed using a laser confocal microscope (Zeiss LSM510, Germany).

### Electrophoretic mobility shift assay (EMSA)

The primers used to amplify the *MdCIbHLH1 *ORF were MdCIbHLH1-zS, 5'-GCGAATTCATGGACGACAGGGAGGAC-3', and MdCIbHLH1-zA, 5'-GCGCGGCCGCCATCATGCCATGGAACCCG-3', containing EcoRI and NotI restriction sites, respectively. The amplified fragment was then inserted into the expression vector *pPICZαA *(Invitrogen, USA) following digestion with EcoRI and NotI. The resultant expression vector was transformed into the yeast strain GS115 following digestion with BstXI. The MdCIbHLH1 protein was prepared according to the manufacturer's instruction manual. The EMSA was carried out according to the manufacturer's instructions (Thermo, USA). The double-stranded oligonucleotides MYC1 (CATTTTACAATTGCTTCGCT), MYC2 (CTCTGGACACATGGCAGATC), MYC3(5) (ACCCCACCATTTGTTAATGC) and MYC4 (ACAATTACAACTGCATGCTT) [9] were used as probes and competitors for the EMSAs. MYC4-mut (ACAATTAACACGTCATGCTT) was used as the mutated competitor.

### ChIP-PCR assay

ChIP analysis was performed using the Chromatin Immunoprecipitation Assay Kit (Millipore, USA) and a modified version of the manufacturer's instructions. Protein and DNA were cross-linked by adding formaldehyde directly to the culture medium to a final concentration of 1%, and the reaction was stopped 10 min later by adding 2 M glycine to a final concentration of 0.125 M. Plant extracts were incubated with GFP antibody (Beyotime, China). The antibody-bound complex was precipitated with protein A-Sepharose beads. The DNA fragments in the immunoprecipitated complex were released by reversing the cross-linking at 65°C for 5 h. Purified immunoprecipitated DNA was first analyzed by PCR and visualized by gel electrophoresis. The primers used for the regular PCR are shown in Additional file 1: Table S1. The following PCR conditions were used: incubation at 95°C for 5 min to activate the polymerase; 35 amplification cycles at 95°C for 20 s, 60°C for 15 s and 72°C for 20 s; and a final extension at 72°C for 10 min. The results are expressed as the ratio to input DNA.

### Genetic transformation of *MdCIbHLH1*

The primers used to amplify *MdCIbHLH1 *cDNA were MdCIbHLH1-fS, 5'-GGATGGACGACAGGGAGGA-3', and MdCIbHLH1-fA, 5'-TGCAATCTACATCATGCCATGG-3'. Subsequently, the PCR product was cloned into the *pMD18-T *vector (TaKaRa, Japan). *MdCIbHLH1 *was double-digested with *Xba*I and *Sal*I and then ligated into the *pBI121 *vector under the control of the *CaMV 35S *promoter. The binary construct *35S::MdCIbHLH1 *was introduced into *Agrobacterium tumefaciens *GV3101, and *Arabidopsis *plants were transformed using the flower dipping method described by [36].

*A. tumefaciens *strain LBA4404 containing either the *35S::MdCIbHLH1:GFP *or *35S::GFP *binary construct was used to transform apple calluses. 'Orin' apple calluses were immersed into *Agrobacterium *suspension cultures for 10 min. The calluses were then co-cultivated in MS media with 1.5 mg l^-1 ^2,4-D and 0.4 mg l^-1 ^BA at 25 ± 1°C in the dark for two days. After co-cultivation, the callus was transferred to MS screening media containing 1.5 mg l^-1 ^2,4-D, 0.4 mg l^-1 ^BA, 100 mg l^-1 ^kanamycin and 250 mg l^-1 ^carbenicillin. The calluses were subcultured four times at 15-day intervals to obtain transgenic calluses. The same binary construct was used for the apple transformation.

Apple leaf segments were excised from shoots grown in vitro 4 weeks after subculture. Leaf strips were immersed into *Agrobacterium *suspension cultures for 10 min and then transferred onto MS media for co-cultivation at 25 ± 1°C in the dark for 2 days. The leaf strips were subsequently transferred to MS media containing 0.15 mg l^-1 ^NAA, 5 mg l^-1 ^6-BA, 10 mg l^-1 ^kanamycin and 250 mg l^-1 ^carbenicillin for regeneration and screening. After adventitious shoots regenerated, they were transferred to MS media containing 0.5 mg l^-1 ^6-BA, 0.1 mg l^-1 ^NAA, 10 mg l^-1 ^kanamycin and 250 mg l^-1 ^carbenicillin for subculturing. The plantlets were used for further investigation.

Young leaves of vigorously growing tobacco were excised into approximately 1 cm × 1 cm strips. Leaf strips were immersed into *Agrobacterium *suspension cultures for 10 min and then transferred onto MS media for co-cultivation at 25 ± 1°C in the dark for 2 days. Subsequently, leaf strips were transferred to MS media containing 0.2 mg l^-1 ^NAA, 3 mg l^-1 ^6-BA, 100 mg l^-1 ^kanamycin and 250 mg l^-1 ^carbenicillin for regeneration and screening. After adventitious shoots regenerated, they were transferred to MS medium containing 1.5 mg l^-1 ^NAA, 150 mg l^-1 ^kanamycin and 250 mg l^-1 ^carbenicillin for rooting. Rooted plantlets were transplanted to soil. After self-pollination, the homozygous transgenic lines were used for further investigation.

### Tolerance analysis of transgenic plants

For the growth measurements of *Arabidopsis*, 7-day-old seedlings were maintained at 4 ± 1°C with a light intensity of 100-150 μmol m^-2 ^sec^-1 ^for 30 days, while at 20 ± 1°C under the same conditions for 7 days as control. The treatment plates were placed vertically with seedlings in the upright position. Three replicates were used for each treatment. Increases in relative root length were measured with a ruler. The relative root length was calculated as the elongation of root length/original root length.

Fifteen-day-old TGC (*35S::MdCIbHLH1:GFP*) and pBIN (*35S::GFP*) calluses (0.5 g) were treated with cold stress. For the cold treatment, the calluses were incubated at 15°C in dark. The control calluses were transferred to MS media and grown for an additional 23 days in dark. The weight increment was measured after this period.

Apple shoot tissue cultures were subcultured on MS nutrient media with 3% sucrose and 0.8% agar for 4 weeks, and the shoot tissue cultures were used to examine cold tolerance. Cold stress was imposed by incubating the cultures at 0 ± 1°C for 7 days with a light intensity of 150 μmol m^-2 ^sec^-1^. The cultures were allowed to recover for 7 days under normal conditions.

Tobacco seedlings were germinated on MS nutrient media with 3% sucrose and 0.8% agar for 15 days, and the seedlings were used to examine chilling and freezing tolerance. Chilling stress was imposed by incubating the seedlings at 4 ± 1°C with a light intensity of 100-150 μmol m^-2 ^sec^-1^. Seedling survival was scored visually after 30 days. For the freezing tolerance assay, the seedlings were cold-acclimated at 4 ± 1°C for 3 days and then frozen at -10°C for 1 to 5 h in dark. The frozen seedlings were thawed overnight at 4°C and transferred back to normal conditions for recovery. Seedling survival was scored visually after 3 days.

Adult tobacco plants grown in potted soil were also used to assess chilling tolerance. Plants were cold-acclimated for 48 h at 15 ± 1°C under a 16-h photoperiod and then exposed to 4°C for 10 h in dark. The plants that underwent the chilling treatment were allowed to recover under normal conditions for 2 h.

For the chilling assay, the proline and MDA (malondialdehyde) contents were measured along with the relative electrolyte leakage content, as previously described [37].

### Western blot protocol and analysis of sumoylation and ubiquitination

For the western blot analysis, TGC calluses were incubated at 15°C in the dark. A total of 2 g of callus material was ground for each sample in a buffer containing 20 mM Tris (pH 7.4), 100 mM NaCl, 0.5% Nonidet P-40, 0.5 mM EDTA, 0.5 mM PMSF and 0.5% protease inhibitor mixture (Sigma, Germany). MdCIbHLH1 protein levels were determined using a protein gel blot with an anti-GFP antibody, as previously described [38].

For the ubiquitination and sumoylation assays, 15-day-old calluses were pretreated with 50 μM MG132 proteasome inhibitor in the dark. Total proteins were extracted as described above. The protein complexes were immunoprecipitated using the Pierce Classic Protein A IP Kit (Thermo, USA) and anti-GFP (Beyotime, China). The resultant proteins were separated with SDS-PAGE and blotted onto a PVDF membrane (Roche, USA). The gel blot was probed with anti-Ub (Sigma, Germany) and anti- SUMO1 (Cell Signaling Technology, USA) antibodies and visualized by chemiluminescence using the ECL plus kit (Trans, China) according to the manufacturer's instructions.

### Statistical analysis

The data are presented as the average of three replicates. SAS (Statistical Analysis System) software (SAS Corporation, Cory, NC, USA) was used for the analysis.

## Authors' contributions

YJH and XMF designed the research and wrote the article. XMF, QZ, LLZ and YQ carried out gene cloning and functional characterization. XBX and HFL participated in EMSA and gene expression analyses. YXY and CXY helped in data analysis and manuscript preparation. All authors read and approved the final manuscript.

## Supplementary Material

Additional file 1 **Table S1**. PCR primers used for ChIP. Click here for file
