# Supplementary material for: The cold-induced basic helix-loop-helix transcription factor gene MdCIbHLH1 encodes an ICE-like protein in apple
Source: BMC Plant Biol. 2012 Feb 15;12:22. doi: 10.1186/1471-2229-12-22 (PMC3352023; doi:10.1186/1471-2229-12-22)
Supplement: Additional file 1 — Table S1. PCR primers used for ChIP. [file 1471-2229-12-22-S1.PDF]

Additional table1

PCR primers used for ChIP

CBF1-S1	TTGAAGGCTCAGTTGGAGAGAG
CBF1-A1	GGTTTAAAGGGTTGGTTTGCG
CBF1-S2	CAGAGATGGCAAGGTTCTTGG
CBF1-A2	CACGTGCCTCATTAACCTGTC
CBF1-S3	TACTCGCCGATGTTTTGATT
CBF1-A3	TTGCTACTCATTGTTACTTGTTCTG
CBF1-S4	CAGACTTGCTACAAATGTAAGGT
CBF1-A4	GTCATGGACTTGTTTGGACCTG
CBF1-S5	CAACAGCAAGTGAGCAACAA
CBF1-A5	CTGGATGACTCACACAAACCT
CBF1-S6	CACACACTTGGCACACACAA
CBF1-A6	CTTTTCGTGGGGATGATTTG
CBF2-S1	GACACACCCTGACAGAAATCG
CBF2-A1	GTACCTATCCTACTGGACTGC
CBF2-S2	CCTCGTACGCCACCAACAGC
CBF2-A2	GAATATGGTTATGTGAAGCATG
CBF2-S3	CTAAGTGCAGGTTTATGAGC
CBF2-A3	CCAGAAAATGCGTTTAAATG
CBF2-S4	GCTCGTCCTCGAGATAAGCC
CBF2-A4	GGCGGCCGGCGTTTGAGG
CBF2-S5	CGAACTCCGGCGACCGTCG
CBF2-A5	CATCACCCCCGATTTGAGTA
CBF2-S6	GACAACCTTGCCAATAATG
CBF2-A6	TATTTTAAACGTACCAATGTAC
CBF3-S1	GCACCTAACCAATACTAGC
CBF3-A1	CGACGAAATATTCCCTAACG
CBF3-S2	GGGTTTCCGAAGGAGTTC
CBF3-A2	GGACCAAATTAGAACTTAG
CBF3-S3	CACATCCGCATCAAAACGTGCC
CBF3-A3	GTTGTACACGGAGTGTGAGG
CBF4-S1	CTGCGTTTTCACGGAAAATG
CBF4-A1	GTGTGAATTGTTGGGTGAGG
CBF4-S2	GTGTGCGGTTCTTCTTGTGC
CBF4-A2	AATTCTGTCATTTTGATCCG
CBF4-S4	ATGTATGAAGTTGTAATT
CBF4-A4	TAAGTTCCTTGCCGTAACAT
CBF4-S3	CCGAGTGCTTCATTATATGTATTC
CBF4-A3	ACCAGGTGGGGAAATGTTGC
CBF5-S1	CAAGGATAAGATTGAGTTTAAG
CBF5-A1	GACGGTTAATAAGTTAACCG

CBF5-S2	GAAGCCCGTTGCAAGACTATG
CBF5-A2	CGGTATTCCCAAACCGAACTG
CBF5-S3	CAAGTTGATCTGACGAAGCTG
CBF5-A3	AAGTGAATGAATCTTGTTGC
CBF5-S4	GGCACCCAGTCCTGCAATTAC
CBF5-A4	CGCAAGTGTGGACAATCTAG
CBF5-S5	GAGACCGATTCCCGTACCG
CBF5-A5	TTAGATGCGCCAGGCTAATC
